# Transcriptional profiling of the leaves of near-isogenic rice lines with contrasting drought tolerance at the reproductive stage in response to water deficit

**DOI:** 10.1186/s12864-015-2335-1

**Published:** 2015-12-29

**Authors:** Ali Moumeni, Kouji Satoh, Ramiah Venuprasad, Rachid Serraj, Arvind Kumar, Hei Leung, Shoshi Kikuchi

**Affiliations:** Rice Research Institute of Iran, Mazandaran Branch, Agricultural Research, Education and Extension Organization (AREEO), PO Box 145, Postal Code 46191-91951 Km8 Babol Rd., Amol, Mazandaran Iran; Plant Genome Research Unit, Agrogenomics Research Center, National Institute of Agrobiological Sciences (NIAS), Kan’non dai 2-1-2, Tsukuba, Ibaraki 305-8602 Japan; International Rice Research Institute, DAPO Box 7777, Metro Manila, 1301 Philippines; Africa Rice Centre (AfricaRice), Ibadan station, c/o IITA, PMB 5320 Oyo road, Ibadan, Nigeria; Agricultural Research (CGIAR ISPC), FAO, Rome, Italy

**Keywords:** Microarray, Gene expression, Leaf profiling, Near-isogenic lines, Water-deficit, *Oryza sativa*

## Abstract

**Background:**

Drought tolerance is a complex quantitative trait that involves the coordination of a vast array of genes belonging to different pathways. To identify genes related to the drought-tolerance pathway in rice, we carried out gene-expression profiling of the leaves of near-isogenic lines (NILs) with similar genetic backgrounds and different set of QTLs but contrasting drought tolerance levels in response to long-term drought-stress treatments. This work will help differentiate mechanisms of tolerance in contrasting NILs and accelerate molecular breeding programs to improve drought tolerance in this crop.

**Results:**

The two pairs of rice NILs, developed at the International Rice Research Institute, along with the drought-susceptible parent, IR64, showed distinct gene-expression profiles in leaves under different water-deficit (WD) treatments. Drought tolerance in the highly drought-tolerant NIL (DTN), IR77298-14-1-2-B-10, could be attributed to the up-regulation of genes with calcium ion binding, transferase, hydrolase and transcription factor activities, whereas in the moderate DTN, IR77298-5-6-B-18, genes with transporter, catalytic and structural molecule activities were up-regulated under WD. In IR77298-14-1-2-B-10, the induced genes were characterized by the presence of regulatory motifs in their promoters, including TGGTTAGTACC and ([CT]AAC[GT]G){2}, which are specific to the TFIIIA and Myb transcription factors, respectively. In IR77298-5-6-B-18, promoters containing a GCAC[AG][ACGT][AT]TCCC[AG]A[ACGT]G[CT] motif, common to MADS(AP1), HD-ZIP, AP2 and YABBY, were induced, suggesting that these factors may play key roles in the regulation of drought tolerance in these two DTNs under severe WD.

**Conclusions:**

We report here that the two pairs of NILs with different levels of drought tolerance may elucidate potential mechanisms and pathways through transcriptome data from leaf tissue. The present study serves as a resource for marker discovery and provides detailed insight into the gene-expression profiles of rice leaves, including the main functional categories of drought-responsive genes and the genes that are involved in drought-tolerance mechanisms, to help breeders identify candidate genes (both up- and down-regulated) associated with drought tolerance and suitable targets for manipulating the drought-tolerance trait in rice.

**Electronic supplementary material:**

The online version of this article (doi:10.1186/s12864-015-2335-1) contains supplementary material, which is available to authorized users.

## Background

Drought is one of the most serious constraints that negatively influences the growth and productivity of rice (*Oryza sativa* L.) and its grain yield potential [1]. Recent climate change research estimates that the water deficit will further deteriorate in years to come [2], and the intensity and frequency of droughts are predicted to worsen [3]. Among cereal crops, lowland-adapted rice genotypes are known to be highly sensitive to the soil WD and evaporative demand, particularly at the reproductive stage [4, 5]. Despite the importance of drought as a constraint, efforts need to be accelerated to develop drought-tolerant rice cultivars. Most of the high-yielding varieties grown in rainfed areas—IR36, IR64, MTU1010, Swarna, Samba Mahsuri, Sabitri, TDK 1—were bred for the irrigated ecosystem and never selected for drought tolerance. In drought years, these varieties have high yield losses, leading to a significant decline in rice production [6]. In the absence of high-yielding good cooking-quality drought-tolerant rice varieties, farmers in the rainfed ecosystem continue to grow these drought-susceptible varieties. Therefore, the improvement of rice’s drought tolerance is considered a promising approach for sustainable production in water-scarce areas [7]. Achieving drought tolerance requires an understanding of the underlying physiological mechanisms and the genetic controls of traits contributing to drought [8]. The mechanisms of the response to WD stress can be studied at the molecular level and at the whole-plant level. Efforts have been made to identify genes and quantitative trait loci related to drought stress in lowland-irrigated rice [1, 9]. However, understanding the molecular basis of manipulating drought tolerance remains a challenge in these varieties.

In the past decade, research on the gene-expression profiling of drought tolerance in rice has primarily relied on the use of a heterogeneous germplasm [10–15]. However, the relationship between genetic variation and drought-tolerant phenotypes may not be immediately clear after WDs were rapidly imposed on heterogeneous drought-stressed and non-stressed germplasms. One promising approach is to use near-isogenic lines (NILs) with similar genetic backgrounds but contrasting levels of tolerance to WDs under long-term drought stress (similar to field conditions) using a dry-down method, which is progressive soil drying measured by the fraction of transpirable soil water (FTSW) as an index of the soil moisture available to plant transpiration and drought intensity. The FTSW shows the total amount of soil water available to support plant-water uptake [1, 16]. NILs are invaluable for testing hypotheses in physiological and genetic studies without any interference from variation in other traits [17]. Two pairs of rice NILs that were previously developed in the genetic background of IR64, in which the two DTNs showed a significantly better performance for grain yield in two lowland and upland environments in different stress and control conditions [18], were used in the present study. The two NILs, IR77298-14-1-2-B-10 and IR77298-5-6-B-18, possessed different sets of QTLs: IR77298-14-1-2-B-10 possessed *qDTY2.2* and *qDTY4.1*, whereas IR 77298-5-6-B-18 possessed only *qDTY4.1*. The QTL *qDTY2.2* showed an effect under severe drought stress with a significantly higher transpiration rate and stomatal conductance, whereas IR77298-5-6-B-18 with constitutively deeper roots, showed an effect under mild to moderate drought stress [18, 19]. Additionally, due to the positive interaction between *qDTY2.2* and *qDTY4.1*, the highest yield advantage under drought has been reported by lines possessing both QTLs [19]. These two pairs of NILs with two major QTLs showed the greatest degree of improvement in grain yield, canopy temperature, the normalized difference vegetation index (NDVI) [19], and increased water uptake ability under drought conditions [20].

The genome-wide identification of the genes regulated by drought [10] allows for a more detailed understanding of the transcriptional response to stress and provides a starting point for the further elucidation of the role of individual genes in the stress response. These studies also help identify putative regulatory elements that are important for functional analysis and crop engineering [14]. Extensive transcriptome analyses of the roots of the two pairs of rice NILs [21] and transcriptome differences between drought tolerance introgression lines, the DT donor, and the drought-susceptible recurrent parent under drought stress [22] have been performed. However, the responses of rice leaves to WD are of greater interest for understanding drought tolerance because leaf growth and development are more sensitive than root growth and development to evaporative demand and soil WD [4, 21, 23] and because the leaves contain the photosynthetic machinery of the plant [24]. If not relieved, WD interrupts reproductive development, inducing premature leaf senescence, wilting, desiccation and death [25]. The majority of the gene-expression profiling studies of rice in response to drought stress [26–28] have investigated single-stress treatment of heterogeneous germplasms at the seedling stage. Currently, little information is available about the leaf gene-expression profiles of lowland-irrigated rice at the reproductive stage under different levels of WD [21]. This research was conducted in a drought at the reproductive stage because 1) rice is highly sensitive to water deficit stress at the reproductive stage, 2) floral fertility is extremely sensitive to water stress, 3) the occurrence of drought at the reproductive stage is more frequent in rainfed drought-prone areas, and 4) the yield loss at the reproductive stage drought is more severe than drought at the seedling or vegetative stage. When stress occurs simultaneously with the irreversible reproductive processes, the molecular analysis of drought tolerance at the reproductive stage is critically important [29]. Here, we report genome-wide expression changes in the leaf transcriptome of two previously developed pairs of rice NILs with different drought tolerances based on grain yield and physiological traits [18], in response to WD treatments at the reproductive stage using Agilent rice oligoarrays (4×44K). Our goal is to study the differential responses of two tolerant NILs to WD stress and to identify putative genes that are responsive to drought and are involved in drought-tolerance mechanisms with the aim of extending our understanding of the genetic mechanisms of drought tolerance in the leaf tissue of rice NILs with a similar genetic background. We hypothesized that the functional classification of a large number of differentially expressed genes (DEGs) generated by microarray experiments in the leaf tissue of the two pairs of NILs would help us identify putative responsive genes and genes with known functions that are involved in drought-tolerance mechanisms. The expression of these genes could be regulated by various transcription factors. We also expected that there might be different mechanisms in responses to WD stress in the two pairs of NILs because they responded differently to WD treatments imposed in this study.

## Results

### Water-deficit treatment and NILs’ physiological traits

In the present study, we examined the effects of two WD treatments on the transcriptome changes and gene-expression profiles of the leaves of two pairs of NILs with contrasting drought tolerances at the reproductive stage. We then tried to analyze the possible link between the microarray profiling with important physiological traits that were previously reported on the same plant materials [18, 20]. The WD treatments started 35 days after seeding (DAS), and the plants were dried until the pot reached the targeted FTSW. We observed from the reported results that among the two +QTLs NILs—including IR77298-14-1-2-B-10 and IR77298-5-6-B-18—that were tolerant to WD treatments, IR77298-14-1-2-B-10 showed a higher relative water content (RWC) [18] and a cooler canopy temperature [20]. It also showed a greater stomatal conductance, a higher transpiration rate, increased water uptake, a higher leaf dry weight, a higher assimilation rate, and a higher grain yield under the WD treatments compared to the susceptible NILs and IR64 (Additional file 1). We found that IR77298-14-1-2-B-10 with two QTLs, i.e., *qDTY2.2* and *qDTY4.1*, showed better performance than IR77298-5-6-B-18 with one QTL, *qDTY4.1*, under severe WD.

In our microarray analysis, we focused on two functional categories of genes: 1) differentially expressed common genes, which reflect drought-responsive genes in the rice NILs and the parent IR64, and 2) putative candidate drought-tolerant genes in the two drought-tolerant NILs (DTN), IR77298-14-1-2-B-10 and IR77298-5-6-B-18, and their corresponding drought-susceptible NILs (DSN) from the same family, IR77298-14-1-2-B-13 and IR77298-5-6-B-11, respectively.

### Global changes in transcripts in response to water deficit

**Fig. 1:**
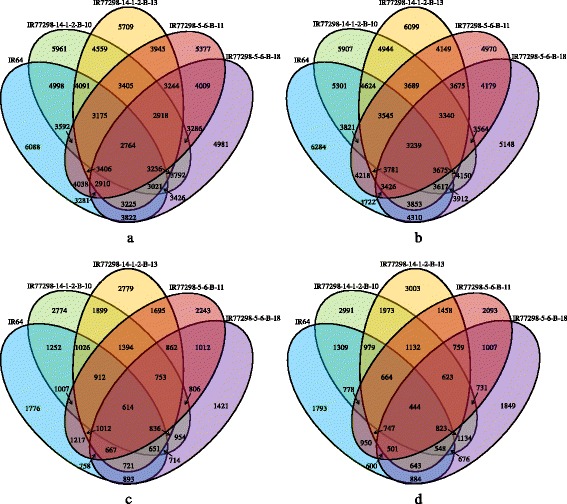
Venn Diagram of differentially expressed genes (DEGs) in leaves of the two pairs of rice NILs and IR64. Number of (**a**) up- and (**b**) down-regulated genes under severe (0.2 FTSW) WD treatment; number of (**c**) up- and (**d**) down- regulated genes under mild (0.5 FTSW) WD treatment

To obtain insight into changes in rice leaf gene-expression profiles under WD treatments and therefore to provide a global overview of the mechanism underlying drought tolerance in the leaf, we examined the effects of two WD treatments, 0.5 and 0.2 FTSW, on the gene-expression profiles of two pairs of rice NILs and the parent IR64 at the reproductive stage, using Agilent rice oligoarrays (4×44K). Fig. 1 shows the number of genes that were differentially expressed, up- and down-regulated, in the leaves of the rice NILs and IR64 under different WD treatments. Overall, a total of 19,033 (43.8 %) transcripts out of 43,494 were differentially expressed in one or both WD treatments, with 16,566 (38.1 %) and 9019 (20.7 %) transcripts up- and down-regulated at 0.2 and 0.5 FTSW between the rice NILs, respectively (adjusted *P* < 0.05). Genes encoding hypothetical proteins were classified as genes of unknown function. Among the 19,033 genes that were differentially expressed, 17.9 % of the genes belonged to the unknown function. A dissection of the gene-expression profiles of the leaves of the rice NILs indicated that the number of DEGs at 0.2 FTSW was higher than that at 0.5 FTSW (2–3 times). The high DTN, IR77298-14-1-2-B-10, displayed a greater number of up- and down-regulated genes (17,633) than other NILs and IR64 during both WD treatments. The number of differentially expressed common genes under severe WD (0.2 FTSW) was also significantly higher in IR77298-14-1-2-B-10 than that under mild WD.

### Functional classification of DEGs in rice NIL leaves influenced by WD treatments

**Fig. 2:**
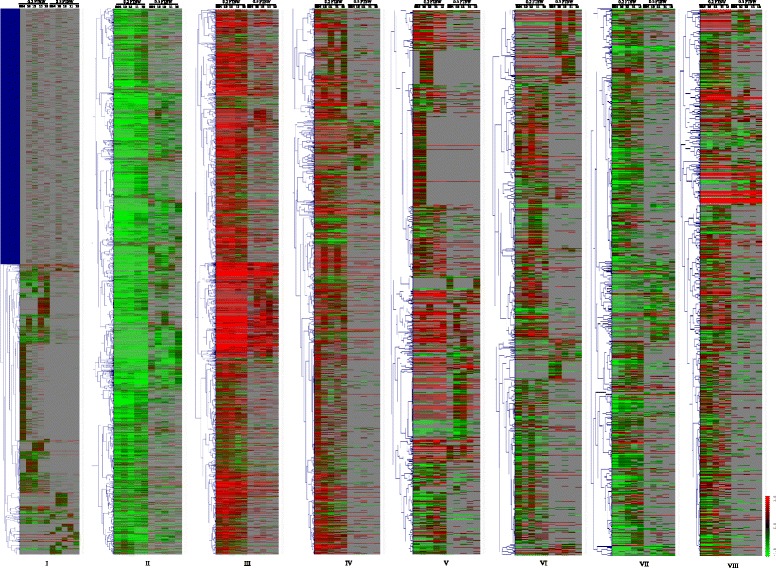
Hierarchical cluster analysis of gene-expression pattern in leaves of five rice genotypes under two water-deficit treatments. The differentially expressed genes under water-deficit treatments with adjusted *P* < 0.05 and −1 ≤ log_2_ratio ≤ +1 (fold change ≥ 2). K-means clustering was performed to identify 8 clusters (I to VIII), each containing various numbers of genes with a similar gene-expression profile under two WD treatments. The numbers are 10 = IR77298-14-1-2-B-10, 13 = IR77298-14-1-2-B-13, 11 = IR77298-5-6-B-11 and 18 = IR77298-5-6-B-18; respectively; 0.2 and 0.5 FTSW are severe and mild WD treatment, respectively. Gene identifiers corresponding to each transcript are from MSU version 6.1 of Rice Oligoarray from Rice Genome Annotation Project (RGAP) 6.1 (http://rice.plantbiology.msu.edu/). A fold change > 2.0 is shown in *red* (up-regulated), a fold change < −2.0 is shown in *green* (down-regulated), and no change is shown in *black* (FDR < 0.05)

Gene ontology (GO) enrichment analysis was conducted to identify the main functional classifications of drought-responsive genes (genes that differentially express commonly in all rice genotypes) through the parametric analysis of gene set enrichment (PAGE) method and genes involved in the drought-tolerance mechanism using the singular enrichment analysis (SEA) method [30]. A relatively large number of drought-responsive genes, including 6003 (36.2 %), 1058 (11.7 %) and 854 (5 %), were differentially expressed common genes in the rice NILs at 0.2 FTSW, 0.5 FTSW and both WDs, respectively (Table 1). We also conducted K-means clustering on the DEGs to analyze the co-expression setting, and eight clusters with coordinated gene-expression profiles were identified (Fig. 2). These clusters reflected the general distribution of gene-expression profiles in leaves in response to WD treatments in different rice genotypes in this study. The complete list of genes in each cluster, including log_2_ratio, can be found in Additional files 2a-h. Cluster I contains 5644 genes with fewer changes in different lines at two WDs with drastically down-regulated genes in IR77298-14-1-2-B-13, whereas clusters II and III contained 3346 and 2877 genes that were highly up- and down-regulated in different lines at severe WDTs, respectively. Clusters IV, V, VI, VII and VIII contained a distribution of 1966, 1658, 1503, 1064 and 975 genes, respectively, with various up-egulated genes in IR77298-14-1-2-B-10 and IR77298-5-6-B-11 and down-regulated genes in IR64, IR77298-14-1-2-B-13 and IR77298-5-6-B-18. These sets of DEGs were subjected to further analysis to investigate the biological functions of the two major groups of DEGs, i.e., differentially expressed common- and specific genes in response to water-deficit treatments. The biological functions of the over-represented drought-responsive genes were obtained by performing a GO analysis (FDR; adjusted *P* < 0.05) of the differentially expressed common genes in the different NILs under the two WDs.

**Table 1:** A summary of the GO enrichment analysis of differentially expressed common genes in the leaves of the NILs of rice under different water-deficit treatments

Water deficit	Expression	Genes	Annotated ID	GO term	Significant GO term/ rice genotypes^§^				
					IR64	10	13	11	18
0.2 FTSW	Up	2764	1813	474	88	62	78	39	71
	Down	3239	2296	453	82	87	53	41	69
	Sub-total	6003	4109	927	170	149	131	80	140
0.5 FTSW	Up	614	424	162	3	5	2	7	3
	Down	444	309	158	27	14	12	11	11
	Sub-total	1058	733	320	30	19	14	18	14
both WD	Up	504	345	149	0(1)^†^	0(1)	0(0)	0(3)	0(1)
	Down	350	251	138	21(24)	24(10)	15(11)	17(11)	13(11)
	Sub-total	854	596	287	21(25)	24(11)	15(11)	17(14)	13(12)

*Up* up-regulated, *down* down-regulated

§Rice NILs 10 = IR77298-14-1-2-B-10; 13 = IR77298-14-1-2-B-13, 18 = IR77298-5-6-B-18; 11 = IR77298-5-6-B-11. The 0.2 and 0.5 FTSW values refer to the severe and mild water-deficit treatments, respectively

†The number of genes that are not bracketed refer to the 0.2 FTSW, and those in brackets refer to the 0.5 FTSW

### Putative functional classifications of drought-responsive genes

**Fig. 3:**
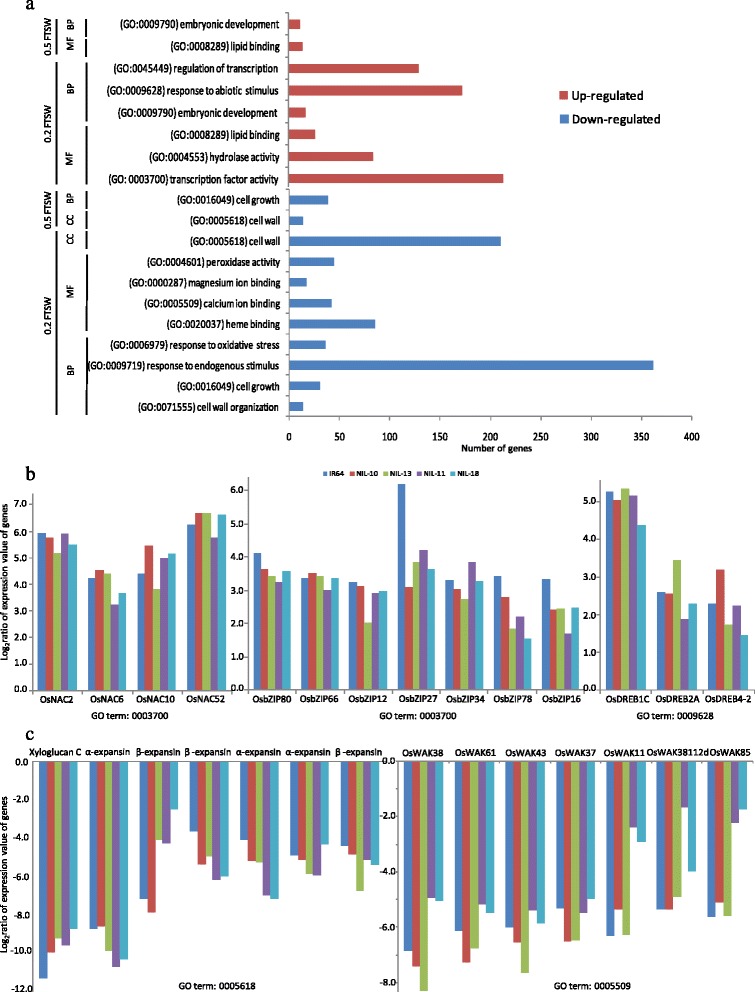
Major GO classifications of the differentially expressed common genes in two pairs of rice NILs and their drought-susceptible parent, IR64. **a** Main GO categories up- and down-regulated at two WD treatments (0.2 and 0.5 FTSW), *CC* cellular components, *MF* molecular functions, and *BP* biological process. **b** Expression profiles of up-regulated selected genes from two main GO terms under severe WD treatment. **c** Expression profiles of down-regulated selected genes from two main GO terms under severe WD treatment. The abreviations are NIL-10 = IR77298-14-1-2-B-10, NIL-13 = IR77298-14-1-2-B-13, NIL-11 = IR77298-5-6-B-11 and NIL-18 = IR77298-5-6-B-18

An analysis of the gene-expression profiles of the differentially expressed common genes indicated that a variety of GO terms related to GO categories such as biological process, cellular components and molecular functions, with the lowest *p*-values, were up- and down-regulated in all rice genotypes in this study under severe, mild and both WD treatments, respectively. Figure 3 summarizes the major GO classifications of the differentially expressed common genes in the rice NILs and IR64. The most prevalent GO terms of up-regulated differentially expressed common genes in rice genotypes in this experiment were a) biological processes: (1) ‘embryonic development’, (2) ‘response to abiotic stimulus’, including heat-shock proteins, dehydration responsive element-binding proteins (DREB), and cytochrome P450s, and (3) the ‘regulation of transcription’; and b) molecular functions, which reflect important aspects of molecular activities, including ‘transcription factor activity’, ‘lipid binding’, ‘hydrolase activity’, and ‘hydrolyzing O-glycosyl compounds’ (Fig. 3a). Genes involved in the major GO classifications of the differentially expressed common genes can be found in Additional file 3. We also observed that a large number of important transcription factor (TF) genes from the MYB, AP2-EREBP, NAC and bZIP families were activated in response to severe WD treatment in the rice NILs (Fig. 3b). More significantly enriched GO terms are shown in Additional file 4. Under mild WD treatment, the GO biological process term ‘embryonic development’ and the molecular function term ‘lipid binding’ were significantly over-represented in the rice NILs. The GO term ‘lipid binding’ was enriched in both WD treatments.

As for the down-regulated differentially expressed common genes, we observed that the GO categories were largely related to growth and signaling systems and electron transport. Details of the GO classifications of down-regulated differentially expressed common genes in all rice NILs and the parent IR64 are shown in Additional file 5. Under severe WD stress, the most significant down-regulated GO terms that were over-represented in all rice NILs were as follows: ‘cell wall organization’, including alpha-expansins and pectinesterases; ‘cell growth’, such as alpha and beta-expansins and potassium transporters; ‘response to endogenous stimulus’ and ‘response to oxidative stress’, for biological processes; ‘heme binding’, including a number of cytochrome P450 and peroxidase gene members; ‘calcium ion binding’, mostly containing OsWAKs, phospholipase Ds; ‘magnesium ion binding’ and ‘peroxidase activity’; and ‘electron carrier activity’, for molecular function. Figure 3c indicates examples of down-regulated genes involved in ‘cell wall’ and ‘calcium ion binding’ in rice NILs and IR64 under severe WD treatment. The GO cellular component ‘cell wall’ was also highly enriched, with several genes. Under mild WD treatment, ‘cell growth’ and ‘cell wall’ were the most significant enriched GO terms for biological processes and cellular components among the rice NILs. Under both WD treatments, the GO terms ‘cell growth’, ‘heme binding’, and ‘cell wall’ were important for biological processes, molecular functions and cellular components, respectively.

### Putative drought-tolerance genes in the two drought-tolerant rice NILs

**Fig. 4:**
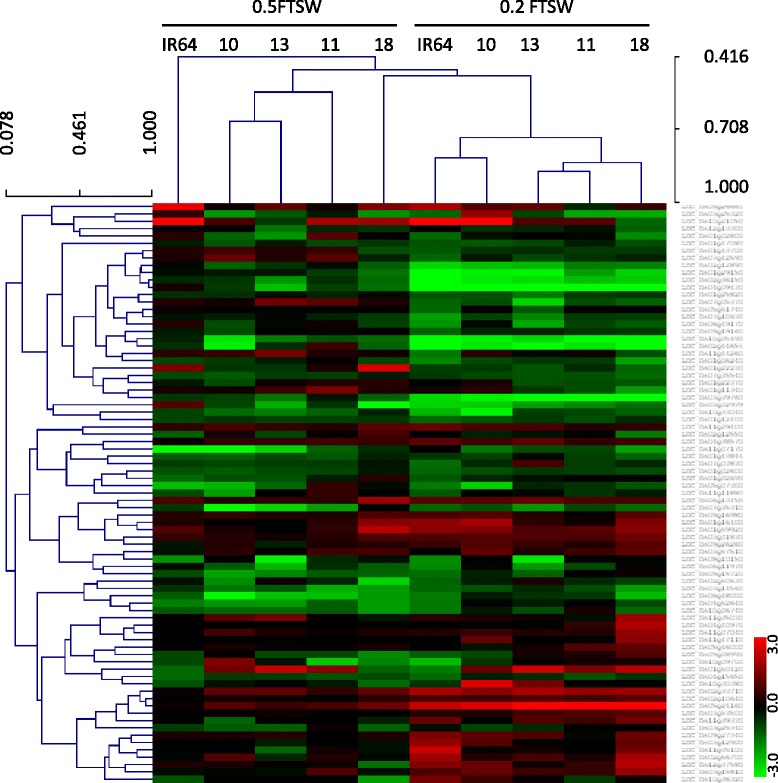
Hierarchical cluster analysis of the differentially expressed specific genes in the leaves of the two DTNs compared to their susceptible counterparts under different WD treatments. In this figure, the heat map displays the expression level of the differentially expressed specific genes in rice NILs and the parent IR64. The numbers are 10 = IR77298-14-1-2-B-10, 13 = IR77298-14-1-2-B-13, 11 = IR77298-5-6-B-11 and 18 = IR77298-5-6-B-18, respectively. 0.2 FTSW and 0.5 FTSW are for leaves under severe and mild WD treatments, respectively. In the color panels, each *horizontal line* represents a single gene, and the color of the line indicates the expression level (in a log scale) of the gene relative to the median in a specific sample: high expression in *red*, low expression in *green*

To identify the putative genes responsible for intrinsic drought tolerance in the two rice DTNs, IR77298-14-1-2-B-10 and IR77298-5-6-B-18, we focused on the genes found to be exclusively differentially expressed in the two DTNs compared to their corresponding DSNs and the parent IR64 in response to the WD treatments. We defined a gene as specifically differentially expressed in the DTNs if it was inversely differentially expressed or if there was no change in expression in its DSN counterpart and IR64. We conducted a cluster analysis on non-redundant DEGs that were specifically expressed in the two DTNs (Fig. 4). The results indicated that the two WD treatments applied in this study were classified into two distinctive clusters. The rice genotypes including DTNs, DSNs and IR64 were also located in separate sub-clusters. We then conducted a GO analysis to convert the specific differentially expressed gene identifiers to standard and interoperable identifiers and to find the main functional categories in terms of biological processes, molecular functions, and cellular components. We then compared the enriched categories between the two DTNs. Table 2 provides an overview of the annotated gene IDs, GO terms and significant GO terms for the differentially expressed specific genes in the two DTNs in response to WD treatments.

**Table 2:** A summary of the GO analysis of differentially expressed specific genes in the leaves of two drought-tolerant NILs of rice under different water-deficit treatments

Situation	Drought Tolerant NILs							
	IR77298-14-1-2-B-10				IR77298-5-6-B-18			
	0.2 FTSW		0.5 FTSW		0.2 FTSW		0.5 FTSW	
	up	down	up	down	up	down	up	down
DESG	495	286	649	688	431	381	274	558
Number of annotated DESG	178	124	271	312	152	147	142	224
GO term	132	117	152	203	123	118	120	146
Significant GO term	29	20	29	71	24	40	12	29
Annotated reference								22,460
Background list								43,494

*FTSW* fraction of transpirable soil water; 0.2 and 0.5 FTSW refer to severe and mild water-deficit treatments, respectively. *Up* up-regulated, *down* down-regulated, *DESG* differentially expressed specific genes in the query list; annotated reference: the number of annotated IDs from the background list; background list: the total number of gene IDs in MSU6.1

We observed that the most significant GO terms for the annotated differentially expressed specific genes in high DTN (IR77298-14-1-2-B-10) that were up-regulated under severe WD were (a) biological processes, including ‘macromolecule localization’, ‘programmed cell death’, ‘protein transport’ and ‘defense response’, (b) molecular function, including ‘ATP binding’, ‘calcium ion binding’, ‘coenzyme binding’, ‘transferase activity, transferring acyl groups’, and ‘hydrolase activity’, and (c) cellular components such as ‘membrane’, ‘thylakoid’, ‘plastid’ and ‘mitochondria’ (Additional file 6). Under mild WD, the significantly up-regulated genes were primarily associated with the ‘metal ion transport’, ‘protein catabolic process’, ‘regulation of transcription’, ‘DNA dependent’, ‘cellular aromatic compound metabolic process’ and ‘regulation of biological quality’ biological processes. The ‘copper ion binding’, ‘transcription regulator activity’ and ‘methyltransferase activity’ molecular functions were also significantly up-regulated under mild WD (Additional file 6).

Under severe WD, down-regulated differentially expressed specific genes had the following significant GO terms: ‘cellular protein metabolic process’ in biological processes and ‘heme binding’, ‘protein binding’, ‘protein kinase activity’, and ‘electron carrier activity’ for molecular functions (Additional file 6). ‘Ribosome’ was the most significantly enriched GO cellular component.

In response to mild WD treatment, the most significant GO biological processes were ‘DNA packaging’, ‘microtubule-based movement’, the ‘phosphorus metabolic process’, and ‘post-translational protein modification’. ‘Microtubule motor activity’, ‘ATP binding’, ‘magnesium ion binding’ and ‘kinase activity’ were the most important enriched GO molecular functions. We found that the GO terms ‘macromolecular complex’, and ‘intracellular organelle’ were also the most important terms of cellular components (Additional file 6).

In the moderate DTN (IR77298-5-6-B-18), the up-regulated candidate genes responsible for drought tolerance under severe WD were classified into ‘transmembrane transport’, ‘RNA processing’, and ‘nucleotide metabolic process’. GO terms for molecular function binding, such as ‘coenzyme binding’, ‘ATP binding’ and ‘transmembrane transporter activity’, were also significantly enriched in the up-regulated genes (Additional file 7). The down-regulated differentially expressed specific genes under mild WD were primarily involved in the ‘catabolic process’, the ‘translation’, the ‘cytoplasmic part’ and the ‘ribosome’, and the molecular functions ‘lyase activity’ and the ‘structural constituent of ribosome’ were also down-regulated. Several of the down-regulated genes under severe WD were associated with ‘protein amino acid phosphorylation’ and the ‘regulation of transcription, categorized as ‘calcium ion binding’, ‘protein kinase activity’ and the ‘transcription of regulator activity’, (Additional file 7). The expression pattern of the down-regulated differentially expressed specific genes under moderate WD was similar to the expression pattern of those under severe WD, except that the genes involved in ‘heme binding’ were the most significantly enriched GO term.

**Fig. 5:**
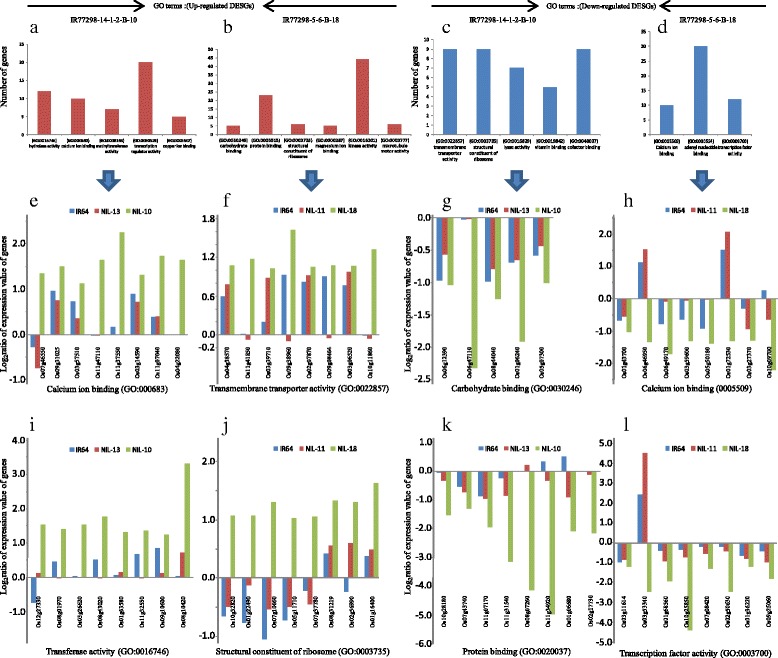
The main differences between two DTNs according to significant GO terms of molecular function category. This figure display differences between the two DTNs, i.e., IR77298-14-1-2-B-10 and IR77298-5-6-B-18, for the main specific GO terms of the molecular function of the differentially expressed specific genes. The GO terms of up-regulated genes are shown in (**a**) IR77298-14-1-2-B-10, (**b**) IR77298-5-6-B-18 and of down-regulated genes are shown in (**c**) IR77298-14-1-2-B-10, (**d**) IR77298-5-6-B-18. (**e-l**) display up- and down-regulated genes of significant GO terms and their expression level (log_2_ratio) of both WD treatments; the abreviations are NIL-10 = IR77298-14-1-2-B-10, NIL-13 = IR77298-14-1-2-B-13, NIL-11 = IR77298-5-6-B-11 and NIL-18 = IR77298-5-6-B-18

We found that the main differences between the two DTNs were related to differentially expressed specific genes involved in ‘calcium ion binding’, ‘transferase activity’, ‘transferring acyl groups’, ‘hydrolase activity, acting on acid anhydrides’, ‘copper ion binding’, ‘transcription regulator activity’ and ‘methyltransferase activity’ in highly DTN, IR77298-14-1-2-B-10, compared with moderate DTN, IR77298-5-6-B-18, for up-regualted genes’ overall WD treatments. In moderate DTN, tolerance could be attributed to ‘transmembrane transporter activity’, the ‘structural constituent of ribosome’, ‘lyase activity’, ‘vitamin binding’ and ‘cofactor binding’ for up-regulated genes (Fig. 5a-b; Additional file 8). In down-regulated differentially expressed specific genes, the high DTN was differentiated from IR77298-5-6-B-18 in ‘carbohydrate binding’, ‘protein binding’, the ‘structural constituent of ribosome’, ‘magnesium ion binding’, ‘kinase activity’ and ‘microtubule motor activity’, with a variety of DEGs in each GO category (Fig. 5c-d; Additional file 9).

We have also checked the genome position of a probe set of differentially expressed specific genes in the two pairs of NILs. We found that 25 out of 79 non-redundant differentially expressed specific genes were located on chromosomes 2, 4, 9 and 10 including 8, 5, 9 and 7 genes, respectively. The remaining differentially expressed specific genes were distributed on chromosomes 1, 3, 5, 6, 7, 8, 11 and 12, with 16, 10, 6, 4, 6, 1, 9 and 2 genes, respectively. Then, we compared the genome positions of differentially expressed specific genes in leaf tissue with the previously reported data in root and leaf tissues on the same rice genotypes. We found that these differentially expressed specific genes were widely located on almost all chromosomes in addition to those previously reported.

Because we found that calcium signaling-related genes were one of the candidates of drought tolerance in high DTN, IR77298-14-1-2-B-10, we analyzed the gene-expression profiles of calcium sensors such as Ca^2+^-dependent protein kinases (CPKs), calcineurin B-like protein-interacting protein kinases (CIPKs), calmodulins (CMLs), protein phosphatase 2C (PP2C), and ABA-responsive genes and ABA-biosynthesis in IR77298-14-1-2-B-10 compared with moderate DTN, IR77298-5-6-B-18. We observed that a higher number of genes related to ABA and the calcium-signaling system in high DTN were differentially expressed compared with medium DTN in this study (Table 3). In our analysis, 131 DEGs were classified as ABA and calcium signaling out of 168 genes in IR77298-14-1-2-B-10. One-hundred and eleven genes were differentially expressed in IR77298-14-1-2-B-10 under 0.2 FTSW, in which 63 were specifically expressed differentially under severe WD treatment, and 48 genes were common to the mild WD treatment. When we compared the signaling-related genes of IR77298-14-1-2-B-10 with those of IR77298-5-6-B-18, we found that 35 genes were specific to the high DTN; 21 were up-regulated DEGs including two ABA-responsive (LOC_Os02g47470, LOC_Os03g18130), three CPKs (OsCPK11, OsCPK24, OsCPK26), four CIPKs (OsCIPK07, OsCIPK14, OsCIPK15, OsCIPK25), eight CMLs (OsCML06, OsCML15, OsCML16 and OsCML19 with distinct expression levels) and four PP2Cs (LOC_Os02g08364, LOC_Os02g55560, LOC_Os03g18970 and LOC_Os09g38550) in the high DTN under severe WD treatment. A total of 14 DEGs from different categories were specifically down-regulated in IR77298-14-1-2-B-10 (Additional file 10).

**Table 3:** The number of up- and down-regulated genes involved in ABA and calcium signaling in leaf tissue of high DTN, IR77298-14-1-2-B-10, compared with moderate DTN, IR77298-5-6-B-18, under different water-deficit treatments

	Drought Tolerant NILs								Total DEGs	Total genes
	IR77298-14-1-2-B-10				IR77298-5-6-B-18					
	0.2 FTSW		0.5 FTSW		0.2 FTSW		0.5 FTSW			
	up	down	up	down	up	down	up	down		
ABA responsive	18	14	14	4	17	13	8	2	38	60
ABA biosynthesis	3	5	4	1	2	3	3	0	10	11
CPKs	7	5	1	1	5	3	0	1	15	30
CIPKs	9	6	9	3	5	3	2	0	19	30
CMLs	12	5	7	1	6	5	2	1	24	37
PP2C	19	8	12	1	15	5	9	3	33	63

Severe and mild water-deficit treatment, according to FTSW, are indicated by 0.2 and 0.5, respectively. *Up* up-regulated, and *down* down-regulated, *CPKs* Ca^2+^-dependent protein kinase, *CIPKs* calcineurin B-like protein-interacting protein kinase, *CMLs* calmudulins, *PPC2* protein phosphatase 2C

### Main differences in molecular functions of the differentially expressed drought-responsive genes in the leaf-root of the rice NILs under a water deficit

To differentiate between the molecular functions of the leaf- and root-expressed genes under severe WD treatment, we conducted a GO analysis of differentially expressed leaf and root genes in the two rice DTNs. Differences were noted in the molecular functions of the up-and down-regulated genes in the leaf and root profiles of the two DTNs (Additional file 11). In the high DTN, IR77298-14-1-2-B-10, the leaf profile was enriched for genes involved in the ‘binding’ GO category, such as ‘ATP’, ‘calcium ion’ and ‘coenzymes’, and ‘catalytic activity’, such as ‘hydrolase’. In the root profile, genes with the molecular functions ‘electron carrier activity’, ‘monooxygenase activity’, and ‘heme binding’ were significantly enriched. For the moderate DTN, IR77298-5-6-B-18, most of the activated differentially expressed specific genes in the leaf were involved in ‘transporter activity’, ‘coenzyme binding’ and ‘ATP binding’, whereas ‘transcription activity’, ‘receptor activity’ and ‘carbohydrate binding’ were important functional groups in the root.

When we analyzed the down-regulated genes of the two DTNs (Additional file 12), ‘heme binding’, ‘carbohydrate binding’, ‘protein binding’ and the ‘structural constituent of ribosome’ were uniquely enriched GO terms in the leaves of high DTN, IR77298-14-1-2-B-10, compared to the roots. For the moderate DTN, the GO terms ‘calcium ion binding’, ‘adenyl nucleotide binding’ and ‘transcription factor activity’ were specific to the leaves.

### Consensus regulatory elements in the promoters of the differentially expressed genes in the two drought-tolerant rice NILs

In silico analysis of 2-kb regions of the promoters of the differentially expressed specific genes in the two rice DTNs in response to WD treatment was performed using RiCES, a Rice *Cis*-Element Searching tool [31]. Motifs with a *lift* value >1.5 and a *p* value < 0.05 in the test dataset appeared to best identify significant relationships between experimental conditions and *cis*-element candidates. Under severe WD, several potential *cis*-acting DNA elements that are common to the transcription factors (TFs) Myb, ACE2, TFIII and SBP were identified in the promoters of the genes that were specifically expressed in the high DTN, IR77298-14-1-2-B-10. DNA elements associated with the TFs MADS(AP1), HD-ZIP, AP2, YABBY and SBP were found in the promoters of the genes expressed in the moderately DTN, IR77298-5-6-B-18. Under mild WD, motifs related to TFs Plant C2H2, WHIRLY and AP2 were shared by the promoters of the genes expressed in IR77298-14-1-2-B-10 and IR77298-5-6-B-18 (Table 4). Regulatory motifs common to HD-ZIP, WHIRLY, HSF, Myb, AP2, VOZ-9 and HSF were identified in the promoters of the down-regulated genes, with VOZ-9 and HSF being specific to severe WD and Forkhead being specific to mild WD treatments. A complete list of putative promoter *cis*-elements and the associated transcription factors in the two DTNs under two WD treatments is shown in Additional file 13. Regulatory motifs that did not match any known sequence were considered to be unknown and novel. We then examined two selected identified *cis*-elements from Table 4 for the two DTNs under severe WD treatment, including (AGATT){2} matched to Myb, and (TGAGTCAG){1,2}, matched to MADS (AP1), using Osiris [32]. The results indicated that most of the detected motifs with (AGATT){2} in this study were for the predicted TF sites of MYBCORE with a *p* value of <10^−3^ that was observed in promoters of 153 genes, including four transcription factors, i.e., NAC (LOC_Os07g48550), GNAT (LOC_Os08g01170), Orphans (LOC_Os10g30880) and AP2-EREBP (LOC_Os12g41060). This NAC gene was up-regulated in highly DTN compared to its DSN. As for (TGAGTCAG){1,2}, matched to MADS (AP1), 535 genes were found with 14 enriched TF sites with a *p* value of <10^−4^ ~ 10^−10^. We also observed vrious genes containing the two tested *cis*-elements in promoter region of DEGs in root tissue of DTNs (Additional file 14).

**Table 4:** Consensus *cis* -regulatory elements of differentially expressed specific genes in two tolerant NILs under different WD treatments

IR77298-14-1-2-B-10				IR77298-5-6-B-18			
Motif	Matching to	Lift	Confidence	Motif	Matching to	Lift	Confidence
Up-regulated							
Severe water-deficit							
(AGATT){2}	Myb	1.89	0.014	(TGAGTCAG){1,2}	MADS(AP1)	1.504	0.038
(CTGACTCA){1,2}	Unkown	1.737	0.045	([TGCA]TTC[TGCA]){3}	Unkown	2.272	0.075
([CT]AAC[GT]G){2}	Myb	2.109	0.005	ATGTCCGTA	Unkown	1.814	0.005
ATGTCCGTA	Unkown	1.52	0.005	CAAT[AT]ATTG	HD-ZIP	1.892	0.054
GACAAGT GGC	Unkown	6.667	0.009	CAAT[TA]ATTG	Unkown	1.892	0.054
GGTACTAACCA	Unkown	12.92	0.005	GACAAGTGGC	Unkown	7.958	0.011
TGACAGTGTCA	ACE2	8.612	0.005	GCAC[AG][ACGT][AT]TCCC[AG]A[ACGT]G[CT]	AP2;YABBY	13.705	0.005
TGGTTAGTACC	TFIIIA	11.48	0.005	TTGTACG[TGCA]A	Unkown	2.548	0.054
T[ACGT]CGTACAA	SBP	2.363	0.05	T[ACGT]CGTACAA	SBP	1.795	0.038
[GA]C[TGCA]T[TC]GGGA[TA][TGCA][TC]GTGC	Unkown	20.67	0.005				
Mild water-deficit							
(C[CA]GTT[GA]){2}	Unkown	3.271	0.006	(AATCT){2}	Unkown	2.747	0.023
ATGTCCGTA	Unkown	2.02	0.006	(C[CA]GTT[GA]){2}	Unkown	3.139	0.006
GCAC[AG][ACGT][AT]TCCC[AG]A[ACGT]G[CT]	AP2;YABBY	7.632	0.003	CAAT[CG]ATTG	Unkown	2.087	0.034
GGCTAATAA	Plant C2H2	2.252	0.018	CAAT[GC]ATTG	HD-ZIP	2.087	0.034
TGACAGTGTCA	ACE2	5.724	0.003	GCAC[AG][ACGT][AT]TCCC[AG]A[ACGT]G[CT]	AP2;YABBY	14.65	0.006
TGACA[ACGT][ACGT][ACGT][ACGT]TGTCA	WHIRLY	2.369	0.006	GTCAAAA[AT]	WHIRLY;WRKY	1.52	0.213
TGACA[TGCA][TGCA][TGCA][TGCA]TGTCA	Unkown	2.369	0.006	TACGGACAT	AP2	1.806	0.006
				TGACA[ACGT][ACGT][ACGT][ACGT]TGTCA	WHIRLY	2.273	0.006
				TGACA[TGCA][TGCA][TGCA][TGCA]TGTCA	Unkown	2.273	0.006
Down-regulated							
Severe water-deficit							
(AATCT){2}	Unkown	1.81	0.015	([ACGT]GAA[ACGT]){3}	HSF	1.882	0.072
(CTGACTCA){1,2}	Unkown	2.045	0.053	([CT]AAC[GT]G){2}	Myb	5.641	0.012
CAAT[AT]ATTG	HD-ZIP	1.866	0.053	ATGTCCGTA	Unkown	8.13	0.024
CAAT[TA]ATTG	Unkown	1.866	0.053	CAAT[CG]ATTG	Unkown	1.823	0.03
GCGT[ACGT]{7}ACGC	VOZ-9	2.705	0.03	CAAT[GC]ATTG	HD-ZIP	1.823	0.03
GCGT[TGCA]{7}ACGC	Unkown	2.705	0.03	GACAAGTGGC	Unkown	4.458	0.006
TGACA[ACGT][ACGT][ACGT][ACGT]TGTCA	WHIRLY	2.997	0.008	GCGT[ACGT]{7}ACGC	VOZ-9	2.151	0.024
TGACA[TGCA][TGCA][TGCA][TGCA]TGTCA	Unkown	2.997	0.008	GCGT[TGCA]{7}ACGC	Unkown	2.151	0.024
TTATTAGCC	Unkown	2.021	0.015	TACGGACAT	AP2	3.786	0.012
TTGTACG[TGCA]A	Unkown	1.795	0.038	TGACA[ACGT][ACGT][ACGT][ACGT]TGTCA	WHIRLY	2.383	0.006
				TGACA[TGCA][TGCA][TGCA][TGCA]TGTCA	Unkown	2.383	0.006
				TTATTAGCC	Unkown	1.607	0.012
Mild water-deficit							
(AGATT){2}	Myb	1.525	0.011	([CG]GCGC[GC]){2}	Fork head	1.593	0.051
([CG]GCGC[GC]){2}	Fork head	1.541	0.049	CAAT[CG]ATTG	Unkown	1.674	0.028
CAAT[CG]ATTG	Unkown	1.979	0.033	CAAT[GC]ATTG	HD-ZIP	1.674	0.028
CAAT[GC]ATTG	HD-ZIP	1.979	0.033	GACAAGTGGC	Unkown	3.41	0.005
GACAAGTGGC	Unkown	2.017	0.003	TACGGACAT	AP2	2.897	0.009
TGACAGTGTCA	ACE2	5.209	0.003	TGACA[ACGT][ACGT][ACGT][ACGT]TGTCA	WHIRLY	1.823	0.005
T[ACGT]CGTACAA	SBP	1.56	0.033	TGACA[TGCA][TGCA][TGCA][TGCA]TGTCA	Unkown	1.823	0.005
				TTGTACG[TGCA]A	Unkown	1.529	0.032

Motif: Examined sequences. Matching to: Destination from built in list of known plant *cis* -element of which the examined motif matches to. Lift, Confidence: Index of association rule analysis

### Validation of transcriptome data

The oligoarray analysis data were validated by quantitative RT-PCR (qRT-PCR) using 9 selected drought-responsive genes from different functional categories with 3 biological replicates of the same set previously reported [21]. We observed that, in most cases, the oligoarray expression patterns of the genes were similar to those generated by qRT-PCR.

## Discussion

The present study provides detailed insight into the response of the leaves of two pairs of rice NILs with contrasting tolerance to drought stress at the whole-genome expression level during the reproductive stage. This work follows an analysis of the grain yield and physiological dissection of drought tolerance [18], a transcriptome analysis of the root [21], genetic, physiological, and gene-expression analyses of yield [20], and an evaluation of the physiological mechanisms contributing to the QTL-combination effects of IR64 NILs, including the two pairs of NILs under drought [19]. We used rice whole-genome Agilent oligoarrays (4×44K) to monitor the transcript profiles of the leaves of two pairs of rice NILs responding to WD stress. Two WD regimes were imposed through a dry-down method [16] to provide long-term drought stress, approximating the field conditions at the reproductive phase, a critical stage at which rice is more sensitive to WD stress and after which grain yield is drastically decreased. Although the results indicated an increased leaf dry weight, leaf area, plant height, assimilation rate, transpiration and canopy temperature, and both +QTLs NILs showed a greater yield under drought than the recurrent parent IR64 (Additional file 1), we did not see significant differences in most traits between DTNs and DSNs, suggesting a role of the +QTL in leaf function rather than leaf characteristics in the DTNs. The same results were reported in previous studies on root and leaf tissues [19, 20]. A better performance of IR77298-14-1-2-B-10 with an interaction between the two QTLs, i.e., *qDTY2.2* and *qDTY4.1*, compared to IR77298-5-6-B-18, with one QTL, *qDTY4.1*, was also reported [20]. Therefore, according to these reported data and the transcriptome profiles of the two DTNs under severe and mild WD treatments, it seems that the effect of *qDTY2.2* is specific to the severe WD stress, whereas *qDTY4.1* showed a greater effect under mild WD stress.

### Transcriptome characterization in the leaves of the rice NILs and the parent IR64

A large number of genes was differentially regulated in the leaves of two pairs of rice NILs and the parent IR64 under severe WD compared to mild WD (roughly 2–3 times more genes, as shown in Fig. 1), suggesting that more genes were affected by this increased stress. The number of drought-responsive genes (both up- and down-regulated) that were differentially expressed under the WD treatments in the leaves of the high DTN (IR77298-14-1-2-B-10), compared to the corresponding susceptible NIL (IR77298-14-1-2-B-13) and IR64, was greater than the other pair of the NILs. Therefore, this high DTN (IR77298-14-1-2-B-10) can be considered more responsive to WD stress. Previous reports have indicated that the number of drought-responsive genes increases with higher levels of stress in rice, such as osmotic stress [33] and drought stresses on the roots [21]. Although these two pairs of NILs were genetically similar to IR64 [18], they showed distinct differences in their gene-expression profiles in response to drought.

### The molecular functions of the putative drought stress adaptive rice genes

A better understanding of the molecular function and quantities of genes that are commonly regulated by WD stress will help identify the mechanism of the adaptive responses to drought in plants. The genes with altered expression are likely those involved in the pathways that underlie plant responses to WD [34]. A GO analysis of the differentially expressed common genes suggests that there is a constant up-regulation of genes such as ‘transcription factors’, ‘lipid binding’ and ‘hydrolase activity’ in both pairs of NILs. Among these regulatory classes, including transcription factors (GO:0003700), a number of genes with promoters containing MYB, AP2-EREBP, NAC and bZIP consensus sequences were over-represented among the activated differentially expressed common genes (Additional file 3). Some of the constantly activated TF genes in this study under severe WD stress, such as *OsNAC2/SNAC1* and *OsNAC6/SNAC2*, were previously reported to significantly enhance drought tolerance in transgenic rice under severe drought stress at the reproductive stage [35] and tolerance to drought, salt, and cold stresses during seedling development [36]. The bZIP TFs TRAB1, OSBZ8, and OsABF1 were also found to be induced by dehydration, salt and drought stress [37, 38]. Furthermore, some of the activated AP2-EREBP members, classes II, III, IV, and OsDREB2A, OsDREB1C and OsDREB4-2 were also reported to be induced by drought and other abiotic stresses [39]. Several TFs belonging to the MYB family play important roles in both stomatal and non-stomatal stress responses by regulating the stomatal number, size and metabolic components under environmental stresses [40]. Various classes of TFs (such as zinc finger, MYB, NAC, and the basic region/Leu zipper motif (bZIP) family transcription factors) protein kinases, protein phosphatases, and enzymes have also been reported to be involved in phospholipid metabolism [41]. Lipid binding-related genes, which were constantly activated in this study, were also found to be induced in response to drought and other abiotic stresses; these factors are thought to play a role as desiccation protectants and have been shown to be involved in protecting macromolecules such as enzymes and lipids [41]. We also observed that a variety of genes related to ‘hydrolase activity’, including hydrolases, alpha-amylases and beta-amylases, were induced in rice NILs in response to severe WD. These gene families are known to play important roles in the stress tolerance of plants through diverse physiological activities [42], and their expression is activated by drought stress in rice. These drought-induced catalytic components are involved in the synthesis or catabolism of drought stress-associated metabolites, and some of these have been used to enhance stress tolerance in transgenic plants [43].

Peroxidases (heme binding) were noted to be down-regulated in the rice NILs in the present study. This may lead to the accumulation of hydrogen peroxide in guard cells and trigger stomatal closure, which is important for reducing water loss and enhancing drought tolerance [44]. Other genes were down-regulated in the rice NILs in response to severe WD, including those involved in calcium signaling, such as calmodulins (CaMs) and calcineurin B-like proteins (CBLs), which have been best characterized as calcium sensors [45]. Furthermore, the down-regulation of genes belonging to the electron carrier activity and photosystem categories (Additional file 5) may be the result of a reduction of photosynthetic activity under drought, which could be due to reductions in stomatal conductance and Rubisco activities, leading to lower carbon fixation, which consequently results in the over-reduction of components within the electron transport chain and the generation of reactive oxygen species [46].

### Different mechanisms of drought tolerance between the two drought-tolerant rice NILs

Through a GO analysis of the differentially expressed specific genes, we were able to differentiate the putative mechanisms and molecular functions of genes involved in drought tolerance in the two DTNs of rice. Under severe WD treatment, 3 GO categories, including ‘calcium ion binding’, ‘transferase activity’ (transferring an acyl group other than amino-acyl groups) and ‘hydrolase activity’ were specifically over-represented in the high DTN, IR77298-14-1-2-B-10, and ‘transmembrane transporter activity’ was over-represented in the medium DTN, IR77298-5-6-B-18 (Fig. 5). We speculate that the following genes may play important roles in the response of IR77298-14-1-2-B-10 to WD stress in [43]: (1) genes involved in calcium signaling, such as CaM-like (CML6) protein, calmodulin-dependent protein kinase (CAMK), protein phosphatase 2C genes such as LOC_Os01g46760, and calcium-binding EF-hand family proteins (Additional file 10), which are key calcium-signaling factors and signal transducers [42, 47, 48]. Ca^2+^ is also critical for the ABA-induced inhibition of stomatal opening, and it is a component in the signaling pathways by which ABA regulates stomatal movements in *A. thaliana* [49] because transpiration rate is primarily controlled by the mechanism of stomata opening and closure. Plants’ adaptation to environmental stresses is dependent upon the activation of vast array of molecular networks involved in stress perception, signal transduction, and the expression of specific stress-related genes and metabolites [42]; (2) hydrolases, which are known to be highly induced in rice in response to drought stress [43]; and (3) drought-induced catalytic components, such as genes related to ‘transferase activity’, which are involved in the synthesis or catabolism of WDS-related metabolites [43]. In the moderate DTN, IR77298-5-6-B-18, the activation of genes in the ‘transmembrane transporters’ category, such as potassium and sulfate transporters, sugar transporters, ATP synthases, and the MATE efflux protein family (Fig. 5f), may be important because they are thought to function in plasma membranes and tonoplasts to adjust the osmotic pressure under stress conditions [41].

Under mild WD stress, we also observed an obvious difference between the two DTNs for different molecular function categories. In IR77298-14-1-2-B-10, genes involved in ‘methyltransferase activity’, ‘transcription factor activity’ and ‘copper ion binding’ were the most significantly enriched functional categories (Fig. 5a, e,i), suggesting that regulatory networks and signaling systems may play important roles in the high DTN under mild WD. In contrast, in IR77298-5-6-B-18, genes related to growth regulatory networks such as the ‘structure of the ribosome’ (Fig. 5b, f, j), ‘lyase activity’ and ‘binding’ (of vitamins and cofactors), were significant. Some members of the bZIP family, such as OsbZIP09 and OsbZIP27, which were activated in this study under mild WD, were also reported to be induced by dehydration stress [50]. The genes in the ‘structure of the ribosome’ category, such as ribosomal proteins (small and large subunits, as shown in Fig. 5j), were found to be activated by drought stress in rice [51]. In general, when the level of WD increased from 0.5 to 0.2 FTSW, the type and number of functional categories also changed for the up-regulated differentially expressed specific genes in the two DTNs.

The two DTNs also showed a difference in the molecular functions of differentially expressed specific genes that were down-regulated under severe or mild WD conditions. Genes involved in cellular metabolic processes and growth, such as ‘carbohydrate binding’, ‘heme binding’, ‘protein binding’ and the ‘structural constituents of the ribosome’ were specifically expressed in IR77298-14-1-2-B-10 (Fig. 5g, k), whereas those genes involved in calcium signaling and regulatory networks, such as ‘calcium ion binding’ and ‘transcription factor activity’ were specifically down-regulated in IR77298-5-6-B-18 under severe WD (Fig. 5h,l). It was previously reported that the repression of metabolic genes during WD stress allows the plant to conserve energy and to subsist on less water [14], conferring better drought tolerance.

The genome positions of the differentially expressed specific genes in the two DTNs were distributed in different chromosomes. Some of these genes were previously reported on chromosomes 2, 4, 8, 9 and 10 in both root and leaf tissues [18–21] and in five genome regions in chromosomes 5, 9, 10, and 12 [20]. This result indicates that in addition to the QTLs identified in DTNs in previous studies, there might be another QTLs within the non-introgressed region, which interacts with the identified QTLs in the introgressed region in the case of drought-stress tolerance.

### Differential expression of the drought-responsive genes in the leaves and roots of the two drought-tolerant rice NILs under water deficit

In this study, we found differences in the differentially expressed genes in the leaves and roots of the DTNs under severe WD stress. The leaf profile was enriched for genes involved in membrane transport and the regulation of various metabolic processes along with hydrolases, which are known to impart stress tolerance to plants by participating in diverse physiological activities [43] and to play a role in plant signaling networks.

The root profile, however, contained many transcripts encoding cytochrome P450 gene family members, the largest category, related to oxidative stress enzymes. Oxygen and heme binding were also reported to increase during long-term drought stress in rice [10, 21]. The differentially expressed specific genes related to ‘carbohydrate binding’, which most of which have the ability to recognize and reversibly bind to well-defined carbohydrate structures in plants, are involved in plant defense [52]. This finding also suggests that the level of carbohydrate metabolism may be reduced during tolerance under drought compared to susceptibility [43]. For the moderate DTN, the activated genes function as transporters and signaling networks in the leaves, whereas in the roots, the transcription factors and the carbohydrate metabolism are more important. Furthermore, a reduction in gene functions, such as transcription factors and signaling systems, was observed in the leaves, whereas a reduction was noted for hydrolases in the roots.

### Significance of the regulatory motifs of the differentially expressed specific genes in the two drought-tolerant rice NILs

The regulatory *cis*-elements present in promoter regions are one of the remarkable aspects of gene regulation, and they provide a detailed explanation for co-expression of a group of co-expressed genes. Under severe WD, the majority of the important regulatory motifs identified in the promoters of the drought-responsive genes in this study were different for the two DTNs, suggesting that different mechanisms may control drought tolerance in the DTNs (Table 4). In the high DTN, the interaction of TFs TFIIIA and Myb with *cis*-elements such as TGGTTAGTACC and ([CT]AAC[GT]G){2} under severe WD may control the stomatal aperture under drought stress [35]. Drought-responsive genes in rice have been reported to contain MYB (C/TAACG/TG) consensus elements [53]. In the medium DTN, putative regulatory motifs such as (TGAGTCAG){1,2}, CAAT[AT]ATTG and GCAC[AG][ACGT][AT]TCCC[AG]A[ACGT]G[CT], which are common to the MADS(AP1), HD-ZIP, AP2 and YABBY TFs, respectively, were identified in the promoter region of the up-regulated differentially expressed specific genes. It was reported that a homeodomain (CAAT[AT]ATTG) associated with a leucine zipper was induced in *Helianthus annuus* by water deficit or abscisic acid. Other studies have indicated that YABBY1 in rice might be involved in the process of the leaf rolling that can occur during drought stress in rice [54]. Under mild WD treatment, more overlap of the *cis*-elements was found between the two DTNs, suggesting a more similar performance of the two DTNs. The information from this study may be useful for further analysis to characterize the putative novel regulatory elements through mutation/deletion analysis to uncover more TFs.

## Conclusions

This study provides a comprehensive overview of the molecular and gene-expression changes under WD treatments in the leaves of rice as photosynthetic machinery using three particular elements: 1) two pairs of rice NILs with high genetic similarity, 2) a 4×44K oligoarray technology covering almost the entire rice genome, and 3) WD treatments via a dry-down method in the reproductive stage, similar to field conditions. This set-up allowed us to carry out transcriptional profiling in the leaf tissue of rice NILs and to identify putative drought-responsive genes and genes involved in tolerance to long-term drought stress. We report here that despite having a similar genetic background, these two pairs of NILs with contrasting drought tolerance have different potential mechanisms/pathways based on the transcriptome data from leaf tissues. The present study provides detailed insight into the gene-expression profiles of rice leaves, including the main functional categories of drought-responsive genes and those that are involved in drought-tolerance mechanisms and potentially affect the plant phenotype in response to water deficits. This work will help breeders improve the drought tolerance of rice cultivars. In the meantime, further work on the physiological aspects of the leaf reactions of rice NILs under drought stress, such as stomatal closure, size and density, will provide useful information.

## Methods

### Plant materials and stress treatments

The rice genotypes used in this study were two pairs of NILs with contrasting yields under drought stress and the parent IR64 [18]. IR77298-14-1-2-B-10 is high-yielding (highly drought-tolerant), whereas IR77298-14-1-2-B-13 is low-yielding under stress (drought-susceptible). Similarly, IR77298-5-6-B-18 is high-yielding (moderately drought-tolerant), whereas IR77298-5-6-B-11 is low yielding under stress (highly drought-susceptible). Experimental procedures such as plant growth, irrigation and stress treatments were performed as previously described for the root transcriptome analysis of the NILs [21].

The following watering regimes were used: (a) control, consisting of well-watered plants and soil kept saturated throughout the experiment, and (b) drought stresses, including two drought-stress conditions of 0.2 FTSW (=20 %) and 0.5 FTSW (= 50 %), where no water was added back to the soil during the dry down. The experimental designs for the WD and control treatment were arranged in a randomized complete block design (RCBD) with 4 replicates.

### RNA isolation

Total RNA was extracted from the uppermost rice leaves, including the flag leaf of the plants of the two pairs of NILs, IR77298-14-1-2-B-10 (highly drought-tolerant), IR77298-14-1-2-B-13 (drought-susceptible), IR77298-5-6-B-18 (moderately drought-tolerant), and IR77298-5-6-B-11 (highly drought-susceptible), subjected to 1.0, 0.5 and 0.2 FTSW. Three replicates at the reproductive stage (a total of 45 independent RNA samples) were extracted using an RNeasy Maxi Kit (Qiagen, Valencia, CA). RNA concentration and quality were tested using a Nanodrop spectrophotometer (ND-1000; Nanodrop Technologies, Wilmington, DE) and a BioAnalyzer G2938A (Agilent Technologies, Santa Clara, CA).

### Oligoarray hybridization and data analysis

A two-dye method was used to directly compare the expression profiles of two samples on the same oligoarray. The probe and array designs were performed using eArray Version 4.5, supplied by Agilent Technologies, and 43,494 probes were selected for this custom array. The probe-arrangement information for the array, Platform No. GPL7252, is available at NCBI GEO [55]. Four sets of 43,494 probes (4×44K microarray formats) were blotted onto a glass slide (25 × 75 mm) at Agilent Technologies in 3 biological replicates.

Cyanine 3 (Cy3)- or cyanine 5 (Cy5)-labeled complementary RNA (cRNA) samples were synthesized from total RNA (850 *ng*) with a low-input RNA labeling kit (Agilent Technologies). Transcriptome profiles specific to the stressed plants were examined by a direct comparison of transcription activities between the stressed condition and non-stressed (control) plants on the same oligoarray. The hybridization solution was prepared with Cy3- and Cy5-labeled cRNA (825 *ng* each) using an in situ Hybridization Kit Plus (Agilent Technologies). The hybridization and washing of the microarray slides were performed according to the manufacturer’s protocols. The slide image files were produced by a DNA microarray scanner (G2505B; Agilent Technologies). The outputs of the oligoarray analysis used in this study (series no. GSE30449) are available at NCBI GEO [55]. All data are MIAME compliant.

The signal intensities of Cy3 and Cy5 were extracted from the image files and normalized to remove any dye effects in signal intensity by rank consistency and the LOWESS method, processed by Feature Extraction Software, version 9.5 (Agilent Technologies). The signal intensities of the oligoarray data were normalized according to the quantile method (global normalization) by EXPANDER version 5.2 [56]. A gene was declared ‘expressed’ if the average signal intensity of the gene was higher than 6 in at least at one condition; otherwise, the gene was defined as not expressed. The genes that were defined as differentially expressed had 1) a log_2_-based ratio (stressed sample/control or non-stressed sample) ≥ 1 or, ≤ –1 and 2) a significant change in gene expression between two plants (*P* ≤ 0.05) using a paired *t*-test (permutation, all; FDR collection, adjusted Bonferroni method). The data processing was performed with MeV version 4.6 [57].

The GO enrichment analysis was performed using log_2_-based ratios of the differentially expressed common genes of all rice NILs through the parametric analysis of the gene set enrichment (PAGE) method and the singular enrichment analysis (SEA) method using agriGO, a GO analysis tool for the agricultural community [30]. A cut-off for the FDR adjusted *P*-value < 0.05 was used to screen the GO term enrichment.

### In silico analysis of the promoters of the expressed genes

The 2-kb upstream sequences (promoters) from the translational start site (ATG) of the rice genes were used to identify highly conserved regions among the promoter sequences of the genes that were differentially expressed (both up- and down-regulated) in the two rice DTNs in response to WD treatments using RiCES, a Rice *Cis*-Element Searcher, through the multiple EM for motif elicitation (MEME) algorithm with a width of 6-8 nucleotides [31]. The presence of the motifs of candidates with a high *lift* value (> 1.0) was evaluated in the 2000-bp upstream region of genes. These motifs were further used for *lift* and confidence value calculation [31]. Motifs with a *lift* value >1.5 in the test dataset appeared to best discriminate significant relationships between experimental conditions and *cis*-element candidates. We set the default threshold of *lift* to 1.5, and the *cis*-element candidates were included in the final candidate list only if their *lift* value was higher than this threshold.

## Additional files

Additional file 1:
**Morphological and physiological traits and grain yield of IR64 introgressed NILs under drought-stress condition.** LDW: leaf dry weight; LA: leaf area; PLH: plant height; GY, grain yield (kg ha-1); TR: transpiration rate; Cond.: stomatal conductance; TWU: total water uptake; A: assimilation rate (photosynthesis); T: transpiration; TTSW: total transpirable soil water; 0.2: severe water stress treatment (FTSW); 0.5: mild water stress treatment (FTSW); 1.0: control. Yellow cell: higher performance of +QTL NIL compared with -QTL NIL. (XLSX 10 kb) Additional file 2:
**The complete list of differentially expressed genes (DEGs) in different clusters in rice genotypes at different water-deficits (clusters I -VIII).** From a to h:10: IR77298-14-1-2-B tolerant NIL, i.e., IR77298-14-1-2-B-10; 13: IR77298-14-1-2-B susceptible NIL, i.e., IR77298-14-1-2-B-13; 18: IR77298-5-6-B tolerant NIL, i.e., IR77298-5-6-B-18; 11: IR77298-5-6-B susceptible NIL, i.e., IR77298-5-6-B-11; 0.2 and 0.5 FTSW are severe and mild water-deficit treatments; blank cell: no change in gene expression. (XLSX 1219 kb) Additional file 3:
**Putative drought-responsive genes, including all significant differentially expressed common genes (DECGs), in the two pairs of NILs and the rice parent IR64 under different water-deficit treatments.** (XLSX 111 kb) Additional file 4:
**GO analysis of up-regulated differentially expressed common genes in the leaves of the rice genotypes including the two pairs of NILs and their drought-susceptible parent in response to different WD treatments.** This figure shows a colorful model of the PAGE analysis generated using agriGO, a web-based gene ontology tool of gene-expression data under the different WD treatments (0.2 and 0.5 FTSW) used in this study. In the figure, the information includes the following: GO term, ontology (including three GO categories: biological process (P), molecular function (F) and cellular component (C)), the number of annotated genes for each GO term, the GO description, a simple colorful model in which the red color system indicates up-regulation and the blue color indicates down-regulation, and different statistical parameters such as z-scores, means and adjusted *P* values (FDR) in the different rice genotypes. (XLSX 1517 kb) Additional file 5:
**GO analysis of the down-regulated differentially expressed common genes in the leaves of the rice genotypes, including the two pairs of NILs and their drought-susceptible parent in response to different WD treatments.** This figure shows a colorful model of the PAGE analysis of gene-expression data under the different WD treatments applied in this study. The information includes the following: GO term, ontology (including 3 GO categories: biological process (P), molecular function (F) and cellular component (C)), the number of annotated genes for each GO term, the GO description, a simple colorful model in which the red color system indicates up-regulation and the blue color indicates down-regulation, and different statistical parameters, such as z-scores, means and adjusted P values (FDR) in the different rice genotypes. (XLSX 1665 kb) Additional file 6:
**All GO classifications as well as the significant GO terms of up- and down-regulated differentially expressed specific genes in IR77298-14-1-2-B-10 in different water-deficit treatments.** C: Cellular Components, F: Molecular Functions, P: Biological Process (XLSX 51 kb) Additional file 7:
**All GO classifications and the significant GO terms of up- and down-regulated differentially expressed specific genes in IR77298-5-6-B-18 in different water-deficit treatments.** C: Cellular Components, F: Molecular Functions, P: Biological Process (XLSX 42 kb) Additional file 8:
**Hierarchical tree graph of the over-represented molecular function GO terms generated by SEA for up-regulated differentially expressed specific genes (DESGs) in the two DTNs in different WD treatments.** The GO terms for severe WD in (a) IR77298-14-1-2-B-10, (b) IR77298-5-6-B-18; the GO terms for mild WD in (c) IR77298-14-1-2-B-10 and (d) IR77298-5-6-B-18. The boxes on the graph represent the GO terms labeled with their GO ID, term definition and statistical information. The significant terms (FDR adjusted *P* ≤ 0.05) are marked in color, whereas non-significant terms are in white. In the diagram, the degree of color saturation of a box is positively correlated with the enrichment level of the term. The solid, dashed, and dotted lines represent two, one and zero enriched terms at the two ends of the line, respectively. (PPT 486 kb) Additional file 9:
**Hierarchical tree graph of the over-represented molecular function GO terms generated by SEA for down-regulated differentially expressed specific genes (DESGs) in the two DTNs under different WD treatments.** GO terms for high DTN, IR77298-14-1-2-B-10, under (a) severe WD and (b) mild WD and for moderate DTN, IR77298-5-6-B-18, under (c) severe WD and (d) mild WD. The boxes on the graph represent the GO terms labeled with their GO ID, term definition and statistical information. Significant terms (FDR adjusted *P* ≤ 0.05) are marked in color, whereas non-significant terms are in white. In the diagram, the degree of color saturation of a box is positively correlated with the enrichment level of the term. The solid, dashed, and dotted lines represent two, one and zero enriched terms at the two ends of the line, respectively. (PPT 696 kb) Additional file 10:
**Different genes involved in ABA and calcium signaling in leaf tissue of rice NILs and the parent IR64 under different water-deficit treatments.** 1: up-regulated; -1: down-regulated; blank: no change in expression; 10: IR77298-14-1-2-B tolerant NIL, i.e., IR77298-14-1-2-B-10; 13: IR77298-14-1-2-B susceptible NIL, i.e., IR77298-14-1-2-B-13; 18: IR77298-5-6-B tolerant NIL, i.e., IR77298-5-6-B-18; 11: IR77298-5-6-B susceptible NIL, i.e., IR77298-5-6-B-11; 0.2 and 0.5 FTSW are severe and mild water-deficit treatments (XLSX 46 kb) Additional file 11:
**The molecular functions of the up-regulated DESGs in the leaves compared to the roots in two DTNs under severe WD.** GO terms for severe WD in the leaves of (a) IR77298-14-1-2-B-10 and (b) IR77298-5-6-B-18; (c) the GO terms for severe WD with the root of IR77298-14-1-2-B-10 and (d) IR77298-5-6-B-18. The boxes on the graph represent the GO terms labeled with their GO ID, term definition and statistical information. The significant terms (FDR adjusted *P* ≤ 0.05) are marked in color, whereas the non-significant terms are shown as white boxes. In the diagram, the degree of color saturation of a box is positively correlated to the enrichment level of the term. The solid, dashed, and dotted lines represent two, one and zero enriched terms at the two ends of the line, respectively. (PPT 565 kb) Additional file 12:
**The molecular functions of down-regulated DESGs in the leaves compared to roots in the two DTNs under severe WD.** GO terms for severe WD in the leaves of (a) IR77298-14-1-2-B-10 and (b) IR77298-5-6-B-18. (c) GO terms for severe WD in the root of IR77298-14-1-2-B-10. (d) GO terms for severe WD in the root of IR77298-5-6-B-18. The boxes on the graph represent the GO terms labelled by their GO ID, term definition and statistical information. The significant terms (FDR-adjusted *P* ≤ 0.05) are marked in color, whereas non-significant terms are shown as white boxes. In the diagram, the yellow-to-red, cyan-to-blue and grayscale represent that the term is activated, repressed or non-significant, respectively. The solid, dashed, and dotted lines represent two, one and zero enriched terms at the two ends of the line, respectively. (PPT 540 kb) Additional file 13:
**List of regulatory motifs in the promoters of the differentially expressed specific genes and their corresponding binding transcription factors in the two drought-tolerant rice NILs under mild and severe water-deficit treatments.** Motif: examined sequences. Matching to: description from the built-in list of known plant *cis*-elements, to which the examined motif matches. ATCIS Info: ATCIS record of AGRIS DB, in which the sequence is (partially) matched to the examined motif. Lift and Confidence: index of association rule analysis. Corresponding TUs: transcription units in which the corresponding region contained the examined motif. (XLSX 119 kb) Additional file 14:
**List of genes with selected motifs and enriched TF sites in the promoter region of the up-regulated DEGs in the leaf and root tissues of the two DTNs under severe WD treatment.** (XLSX 305 kb)
