# Supplementary figures and images for: Transcriptional profiling of the leaves of near-isogenic rice lines with contrasting drought tolerance at the reproductive stage in response to water deficit
Source: BMC Genomics. 2015 Dec 29;16:1110. doi: 10.1186/s12864-015-2335-1 (PMC4696290; doi:10.1186/s12864-015-2335-1)

## Slide 1
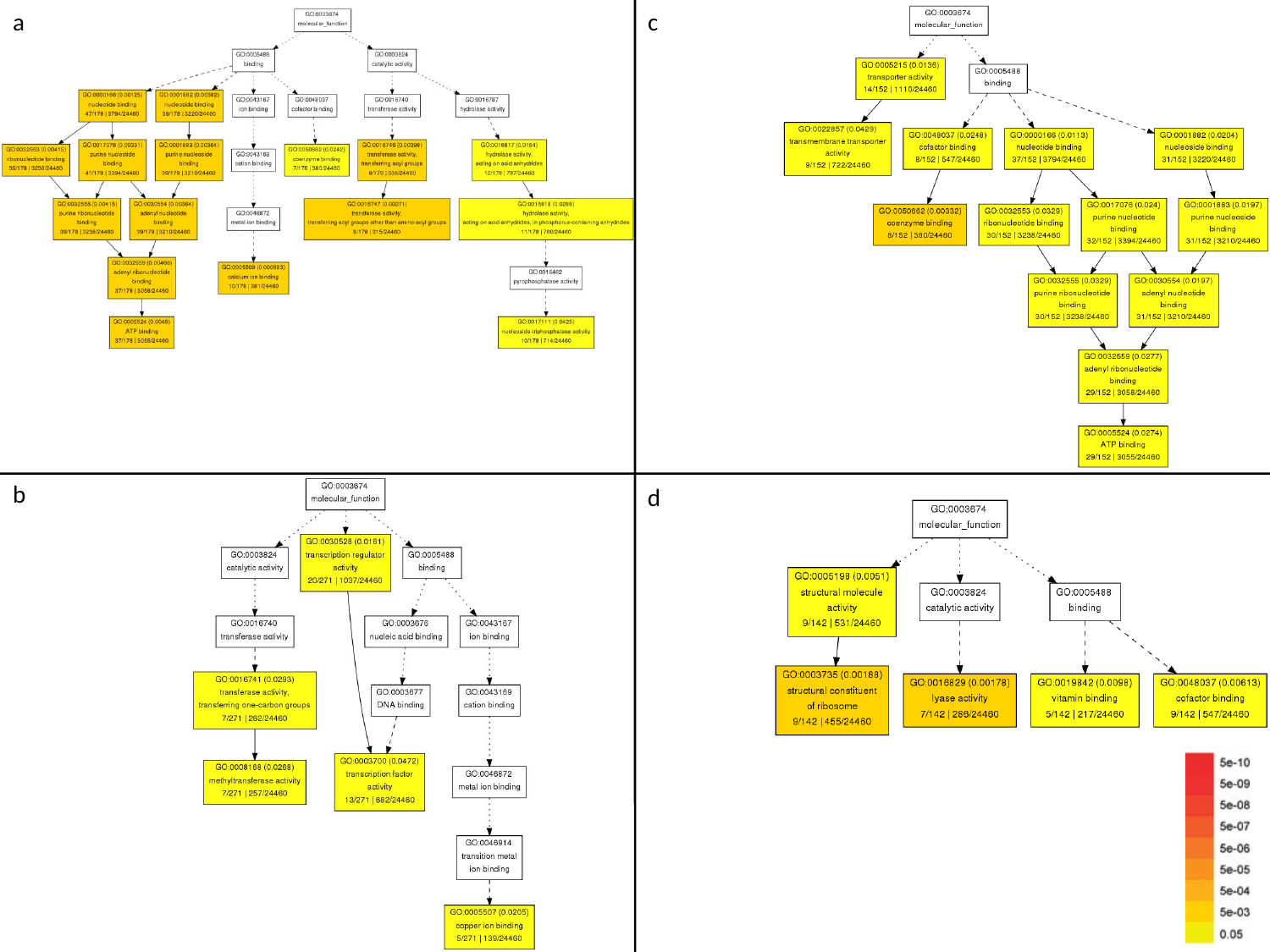

a
c
b
d

Supplement: Additional file 8: — Hierarchical tree graph of the over-represented molecular function GO terms generated by SEA for up-regulated differentially expressed specific genes (DESGs) in the two DTNs in different WD treatments. The GO terms for severe WD in (a) IR77298-14-1-2-B-10, (b) IR77298-5-6-B-18; the GO terms for mild WD in (c) IR77298-14-1-2-B-10 and (d) IR77298-5-6-B-18. The boxes on the graph represent the GO terms labeled with their GO ID, term definition and statistical information. The significant terms (FDR adjusted P ≤ 0.05) are marked in color, whereas non-significant terms are in white. In the diagram, the degree of color saturation of a box is positively correlated with the enrichment level of the term. The solid, dashed, and dotted lines represent two, one and zero enriched terms at the two ends of the line, respectively. (PPT 486 kb) [file 12864_2015_2335_MOESM8_ESM.ppt]

## Slide 1
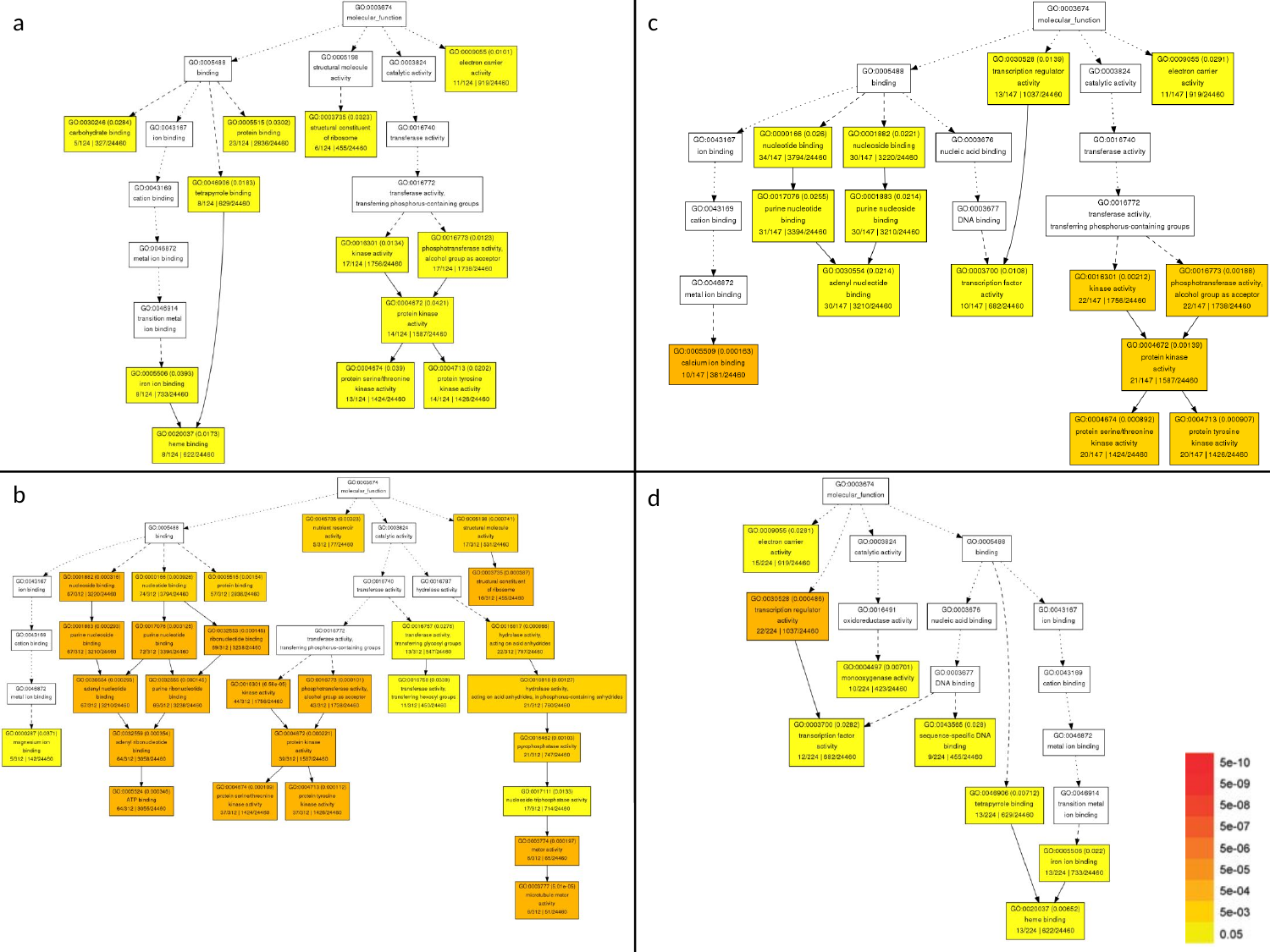

a
c
b
d

Supplement: Additional file 9: — Hierarchical tree graph of the over-represented molecular function GO terms generated by SEA for down-regulated differentially expressed specific genes (DESGs) in the two DTNs under different WD treatments. GO terms for high DTN, IR77298-14-1-2-B-10, under (a) severe WD and (b) mild WD and for moderate DTN, IR77298-5-6-B-18, under (c) severe WD and (d) mild WD. The boxes on the graph represent the GO terms labeled with their GO ID, term definition and statistical information. Significant terms (FDR adjusted P ≤ 0.05) are marked in color, whereas non-significant terms are in white. In the diagram, the degree of color saturation of a box is positively correlated with the enrichment level of the term. The solid, dashed, and dotted lines represent two, one and zero enriched terms at the two ends of the line, respectively. (PPT 696 kb) [file 12864_2015_2335_MOESM9_ESM.ppt]

## Slide 1
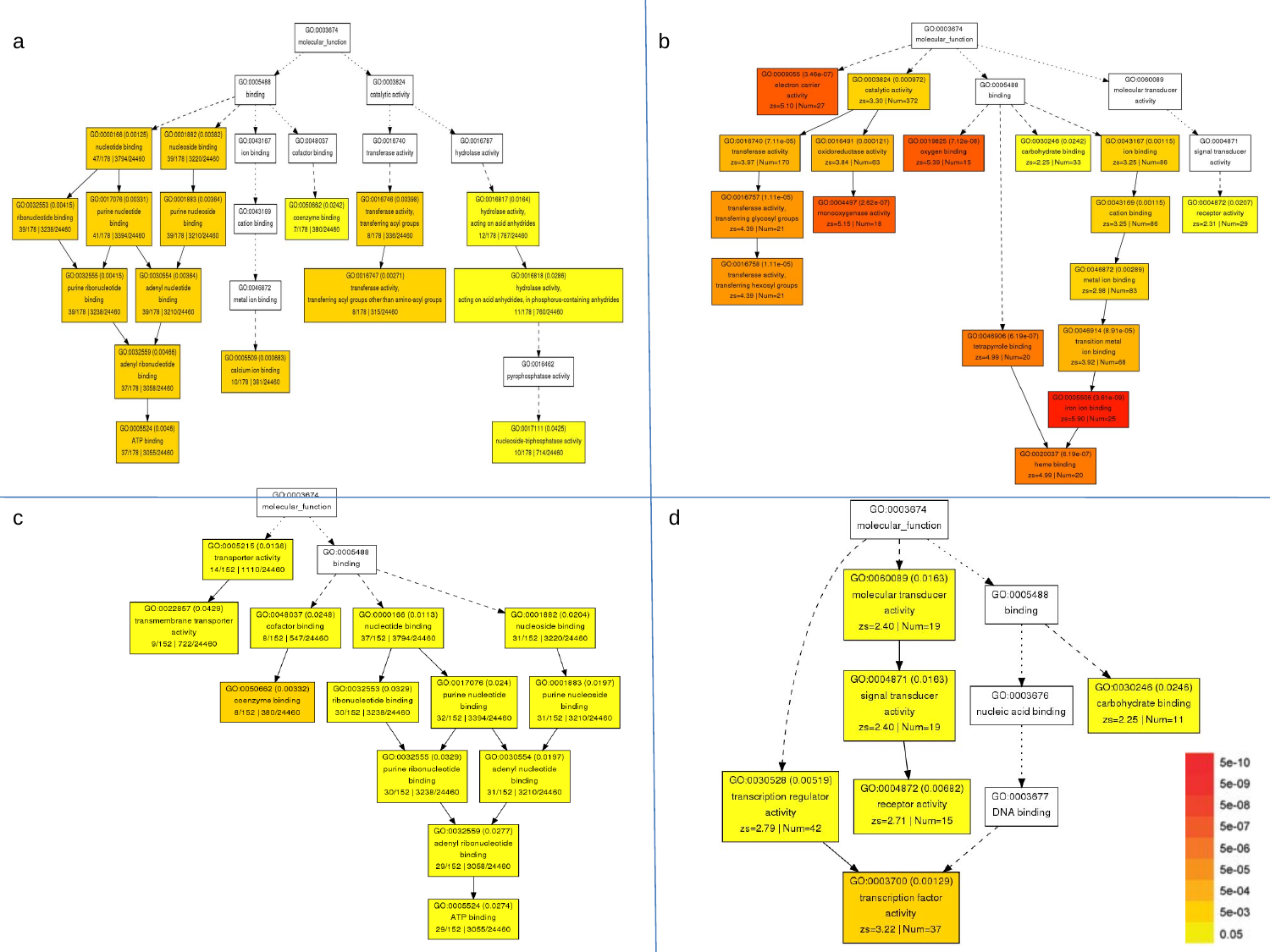

a
b
c
d

Supplement: Additional file 11: — The molecular functions of the up-regulated DESGs in the leaves compared to the roots in two DTNs under severe WD. GO terms for severe WD in the leaves of (a) IR77298-14-1-2-B-10 and (b) IR77298-5-6-B-18; (c) the GO terms for severe WD with the root of IR77298-14-1-2-B-10 and (d) IR77298-5-6-B-18. The boxes on the graph represent the GO terms labeled with their GO ID, term definition and statistical information. The significant terms (FDR adjusted P ≤ 0.05) are marked in color, whereas the non-significant terms are shown as white boxes. In the diagram, the degree of color saturation of a box is positively correlated to the enrichment level of the term. The solid, dashed, and dotted lines represent two, one and zero enriched terms at the two ends of the line, respectively. (PPT 565 kb) [file 12864_2015_2335_MOESM11_ESM.ppt]

## Slide 1
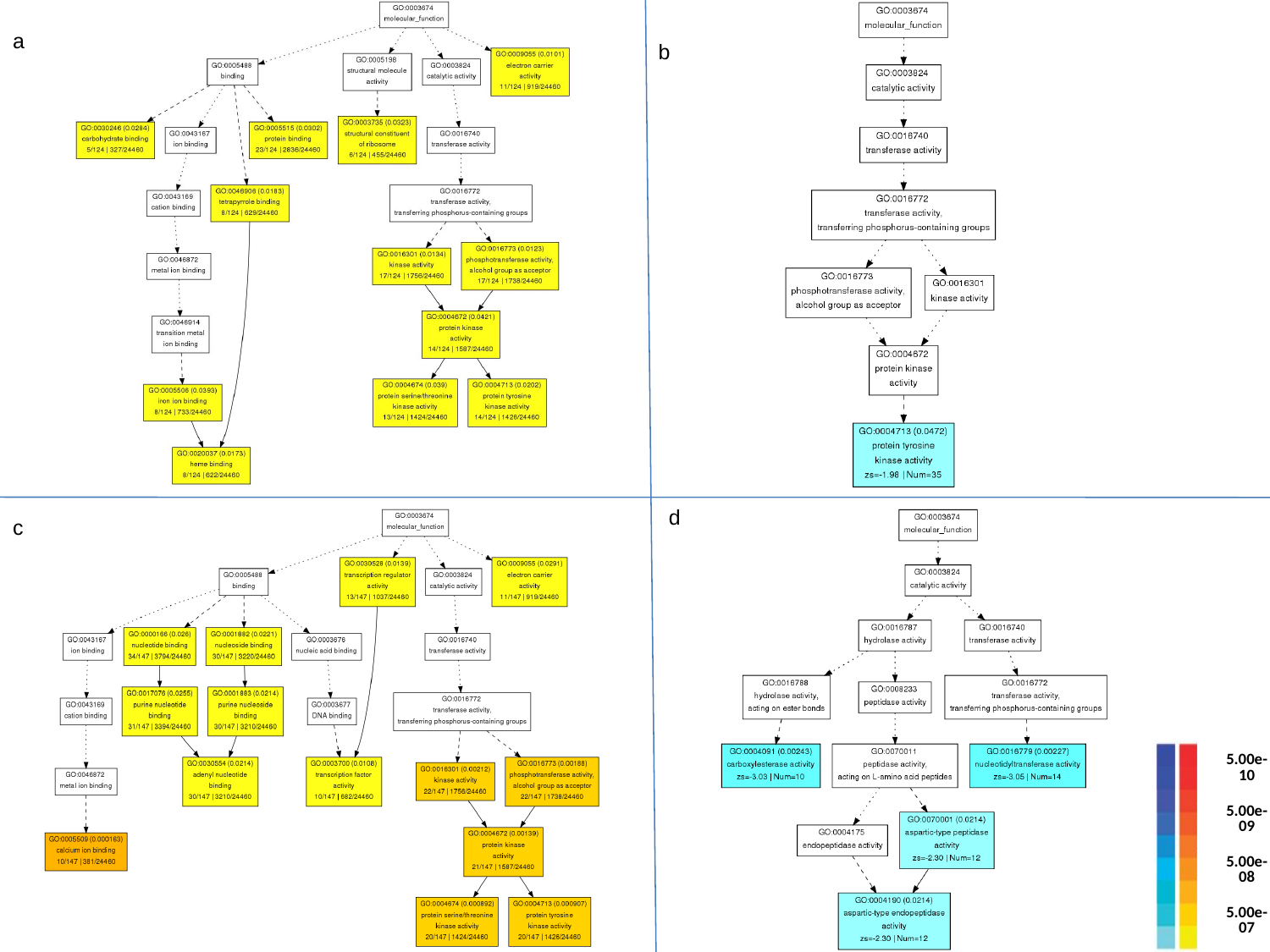

a
b
d
c
| | | 5.00e-10 |
| --- | --- | --- |
| | | 5.00e-09 |
| | | 5.00e-08 |
| | | 5.00e-07 |
| | | 5.00e-06 |
| | | 5.00e-05 |
| | | 5.00e-04 |
| | | 5.00e-03 |
| | | 5.00e-02 |

Supplement: Additional file 12: — The molecular functions of down-regulated DESGs in the leaves compared to roots in the two DTNs under severe WD. GO terms for severe WD in the leaves of (a) IR77298-14-1-2-B-10 and (b) IR77298-5-6-B-18. (c) GO terms for severe WD in the root of IR77298-14-1-2-B-10. (d) GO terms for severe WD in the root of IR77298-5-6-B-18. The boxes on the graph represent the GO terms labelled by their GO ID, term definition and statistical information. The significant terms (FDR-adjusted P ≤ 0.05) are marked in color, whereas non-significant terms are shown as white boxes. In the diagram, the yellow-to-red, cyan-to-blue and grayscale represent that the term is activated, repressed or non-significant, respectively. The solid, dashed, and dotted lines represent two, one and zero enriched terms at the two ends of the line, respectively. (PPT 540 kb) [file 12864_2015_2335_MOESM12_ESM.ppt]
